# Foundation models for cardiovascular disease detection via biosignals from digital stethoscopes

**DOI:** 10.1038/s44325-024-00027-5

**Published:** 2024-10-11

**Authors:** George Mathew, Daniel Barbosa, John Prince, Subramaniam Venkatraman

**Affiliations:** Eko Health, Emeryville, CA USA

**Keywords:** Arrhythmias, Heart failure

## Abstract

Auscultation of the heart and the electrocardiogram (ECG) are two central components of the cardiac exam. Recent innovations of the stethoscope have enabled the simultaneous acquisition of a high-quality digital acoustic signal and ECG. We present foundation models trained on phonocardiogram (PCG) and ECG data collected from digital stethoscopes during routine clinical practice. We show that these foundation models that are pre-trained on large unlabeled datasets in a self-supervised manner can be fine-tuned for a variety of cardiovascular disease detection tasks. This is the first study that builds foundation models specifically for synchronously captured PCG and ECG data. Our approach is based on the recently developed masked autoencoder framework which we extend to handle multiple signals that are synchronously captured. This paradigm makes it possible to use large capacity models leading to superior performance even though the size of datasets with medical label annotations may be limited.

## Introduction

Foundation models^1^ have made immense strides in numerous domains including language modeling^2,3^, computer vision^4,5^ and speech recognition^6^. In this work, we describe foundation models for detection of cardiovascular disease states based on phonocardiogram (PCG) and electrocardiogram (ECG) data collected from digital stethoscopes during regular clinical practice. As per the definition of foundation models, these models are pre-trained in a self-supervised fashion on large unannotated datasets and then adapted/fine-tuned for a variety of downstream disease detection tasks. In this paper, we demonstrate such models being adapted for heart murmur detection, atrial fibrillation detection and low-ejection fraction detection and discuss other potential applications as well.

Heart sounds from stethoscopes and ECGs play a fundamental role in modern clinical practice. Machine-learning based algorithms have been developed to automatically detect murmurs associated with Structural Heart Disease (SHD) from heart sounds^7–12^. Similarly, machine-learning algorithms have been used with ECGs to detect a wide spectrum of abnormalities from arrhythmias^13^ to cardiac contractile dysfunction^14^. So far, supervised learning using convolutional neural networks have been the dominant paradigm for these problems. The primary limitation with these approaches has been the relatively small model capacities that could possibly be used due to constraints on the amount of annotated data to facilitate the supervised training. In this work, we leverage the latest self-supervised techniques to pre-train foundation models on large unannotated datasets. This facilitates the creation of larger capacity models that can then be fine-tuned for various cardiovascular disease detection tasks resulting in better performance.

Transformer models based solely on the attention mechanism were introduced in ref. ^15^ and soon after, foundation models were built based on transformer blocks - BERT^2^ (using masked language modeling) and GPT^16^ (based on autoregressive language generation) being the primary examples. These approaches that proved immensely successful in the language domain eventually made inroads into both audio^17^ and vision^18^ tasks. The Vision Transformer model^18^ was one of the first pure transformer models applied directly to images. Initial implementations of the Vision Transformer (ViT) were based on supervised learning. However recently, Vision Transformer models have been adapted to build foundation models where they are pre-trained using a masked autoencoding approach^5^ (echoing the masked language modeling approach used for BERT) and then fine-tuned for image recognition tasks. Similar models also work for audio classification tasks where instead of images, the model is applied on the spectrogram representations of the audio^19^.

The work described in this paper is one of the first instances of such techniques being applied to cardiac signals (PCG and ECG). We apply the masked autoencoding approach described in ref. ^5^ individually to PCG and ECG signals. Additionally, we extend the approach to work with synchronously captured PCG and ECG signals (See Fig. 9). To the best of our knowledge, this is the first adaptation of the masked autoencoder approach in ref. ^5^ to multiple signals with different modalities and more specifically to synchronously captured signals with different modalities. Pre-training models that can process synchronously captured ECG and PCG signals in this fashion allows the model to learn patterns in the timing of electrical activities of the heart relative to the timing of mechanical activities of the heart (as manifested in the heart sounds via PCG). Thereby, in addition to the features coming from the individual PCG/ECG signals, features related to relative timings of electrical and mechanical activity are likely to be learned by the model and be useful for disease state detection.

The paradigm of foundation models can be particularly powerful for bio-medical applications because of the time and cost investments required to create high-quality curated datasets with medical label annotations. If unlabeled data sources are available, one can utilize them to pre-train foundation models that are capable of generating high-quality general representations of the underlying data. This negates the need for prohibitively large annotated datasets to be able to obtain the best performance from large models. Instead, small datasets of few hundreds or thousand participants with associated medical labels suffice to fine-tune the foundation model for the relevant disease detection task.

In related work, Vision Transformer models have been used for detection of arteriovenous fistula (AVF) stenosis from the blood flow sounds captured by a digital stethoscope^20^. This approach was based purely on supervised training. In ref. ^20^, the authors preprocessed the audio data into two-dimensional image presentations which were fed into a ViT model. One of the image representations of sound used in that study was a mel-scaled, decibel (dB)-scaled spectrogram. A spectrogram depicts the spectrum of frequencies of a signal as it varies with time. The x-axis represents time, the y-axis represents frequency, and amplitude of a particular frequency component at a given point in time is represented by the intensity of color for the image representation. The spectrograms were generated from the AVF blood flow sound signals using short-time Fourier transforms. A fast Fourier transform was applied on short windows which were shifted by an amount (equal to the hop length). The mel-scaled dB-scaled spectrograms were generated by logarithmic rescaling of the amplitude and frequency axis. The amplitude was converted to the dB scale and the frequency axis was transformed onto the Mel scale. Once the mel-spectrograms were generated, they were treated as single-channel images which were input to the ViT model.

There are numerous prior studies where self-supervised approaches have been applied for biosignals. For examples of self-supervised approaches applied to 12-lead ECG signals, see refs. ^21–29^. For examples of self-supervised approaches applied to electroencephalogram (EEG) signals, see refs. ^30–32^. The dominant pre-training method in these prior studies is contrastive learning which is usually based on the Siamese network architecture in conjunction with a contrastive loss so that the model is trained to minimize the distance between similar pairs of inputs and maximize the distance between dissimilar pairs^33–35^.

More recently, self-supervised approaches have been applied for signals collected from wearable devices^36–38^. In ref. ^38^, foundation models were built for two biosignals: photoplethysmography (PPG) and electrocardiogram (ECG) recorded from the Apple Watch as part of a large longitudinal Apple Heart and Movement Study (AHMS). For the work in ref. ^38^, the models analyzed the PPG and ECG signals individually and the pre-training method was based on contrastive learning.

Machine learning approaches for health acoustics is an active area of research. For examples of machine learning applied to heart sounds, see refs. ^7–12^. In other health acoustic studies, algorithms have been developed to identify coughers^39^, to detect COVID-19 from breath and cough audio^40–42^ and to detect tuberculosis^43–45^. Most of the prior work applying machine learning to health acoustics have been based on fully supervised learning frameworks. However, very recently, in ref. ^46^, a team from Google Research described a Health Acoustic Representations (HeAR) model (which used a self-supervised framework based on a masked autoencoding approach) that evaluates cough and breath sounds with a focus on downstream tasks like COVID-19 and tuberculosis detection. Our work applies a similar approach for PCG and ECG signals with a focus on detection of cardiovascular diseases and in particular adapts the approach to analyze synchronously captured PCG and ECG signals.

All PCG and ECG data used for this work were captured using Eko Digital Stethoscopes. The Eko Core digital stethoscope captures PCG signals only. The Eko Duo digital stethoscope captures both PCG and a single lead ECG synchronously. When the stethoscopes are paired with an Eko Mobile application, clinicians can make recordings of PCG and ECG data that are saved in a Health Insurance Portability and Accountability Act (HIPAA) compliant database (Eko cloud database). Technical characteristics of the signals recorded and the filters applied before analysis are listed in Table 1. Both the Eko Core (https://www.accessdata.fda.gov/cdrh_docs/pdf20/K200776.pdf) and Eko Duo (https://www.accessdata.fda.gov/cdrh_docs/pdf17/K170874.pdf) digital stethoscopes are FDA-cleared medical devices that have undergone rigorous testing to verify compliance with the IEC 60601 standard for medical equipment.

**Table 1:** Technical characteristics of PCG and ECG data from Eko digital stethoscopes

PCG	
Sampling rate	4000 Hz
Resolution	16 bit
PCG sensor technology	Digital MEMS microphone
Filters applied	High-pass filter (30 Hz cutoff)
	Low-pass filter (800 Hz cutoff)

ECG	
Sampling rate	500 Hz
Resolution	16 bit
ECG electrode material	Stainless steel
ECG electrode spacing	45–55 mm
Filters applied	50/60 Hz mains filter
	Baseline wander removal
	Savitzky–Golay smoothing filter

## Results

For all our experiments, we use a modified version of the “base” model described in ref. ^5^ and the associated code-base (https://github.com/facebookresearch/mae). The parameters for this modified “base” model are listed in Table 2. This is found to be the best performing model for the fine-tuning tasks described in this Section. For all the classification tasks, we show the Receiver Operating Characteristic (ROC) curves and the primary endpoints are Sensitivity, Specificity and Area Under the ROC Curve (AUC) which captures the overall accuracy of the classifier. For details on the pre-training and fine-tuning phases, see the Methods section. We present results for models trained on individual signals (PCG or ECG), as well as the multi-signal variant of the model that analyzes synchronous PCG and ECG. For pre-training each model, we use large unlabeled datasets of recordings captured by users of Eko digital stethoscopes during regular clinical practice. Clinical users of Eko digital stethoscopes, when interested, can pair their stethoscopes with an Eko Mobile application and make recordings of PCG and/or ECG data that are saved in the Eko cloud database. These recordings have neither medical labels nor personally identifiable information associated with them. It is this large dataset of unlabeled recordings that form the basis for pre-training each model. The pre-training settings and dataset sizes used for each of the models are listed in Table 7. All experiments are executed on a system with 8 NVIDIA A100 Tensor Core GPUs with 40GB memory.

**Table 2:** Parameters for the modified “base” Masked AutoEncoder model

Encoder	
No. of transformer layers	12
Transformer embedding dimension	768
Feed-forward dimension	3072
Number of attention heads	12
Number of trainable parameters	85,254,144

Decoder	
No. of transformer layers	4
Transformer embedding dimension	384
Feed-forward dimension	1536
Number of attention heads	6
Number of trainable parameters	7,492,864
Total number of trainable parameters: 92,747,008	

We note that there is no overlap between the datasets used for pre-training and fine-tuning each model. When fine-tuning a pre-trained model for a classification task, we perform a parameter sweep for a small set of hyper-parameters (label-smoothing, masking-ratio and number of final encoder layers unfrozen) to obtain the best model which is selected based on the AUC for the classification task on the validation dataset. The range of values swept over for each hyper-parameter and the optimal values for each hyper-parameter are listed for each classification task in Table 8.

### PCG model

**Fig. 1:**
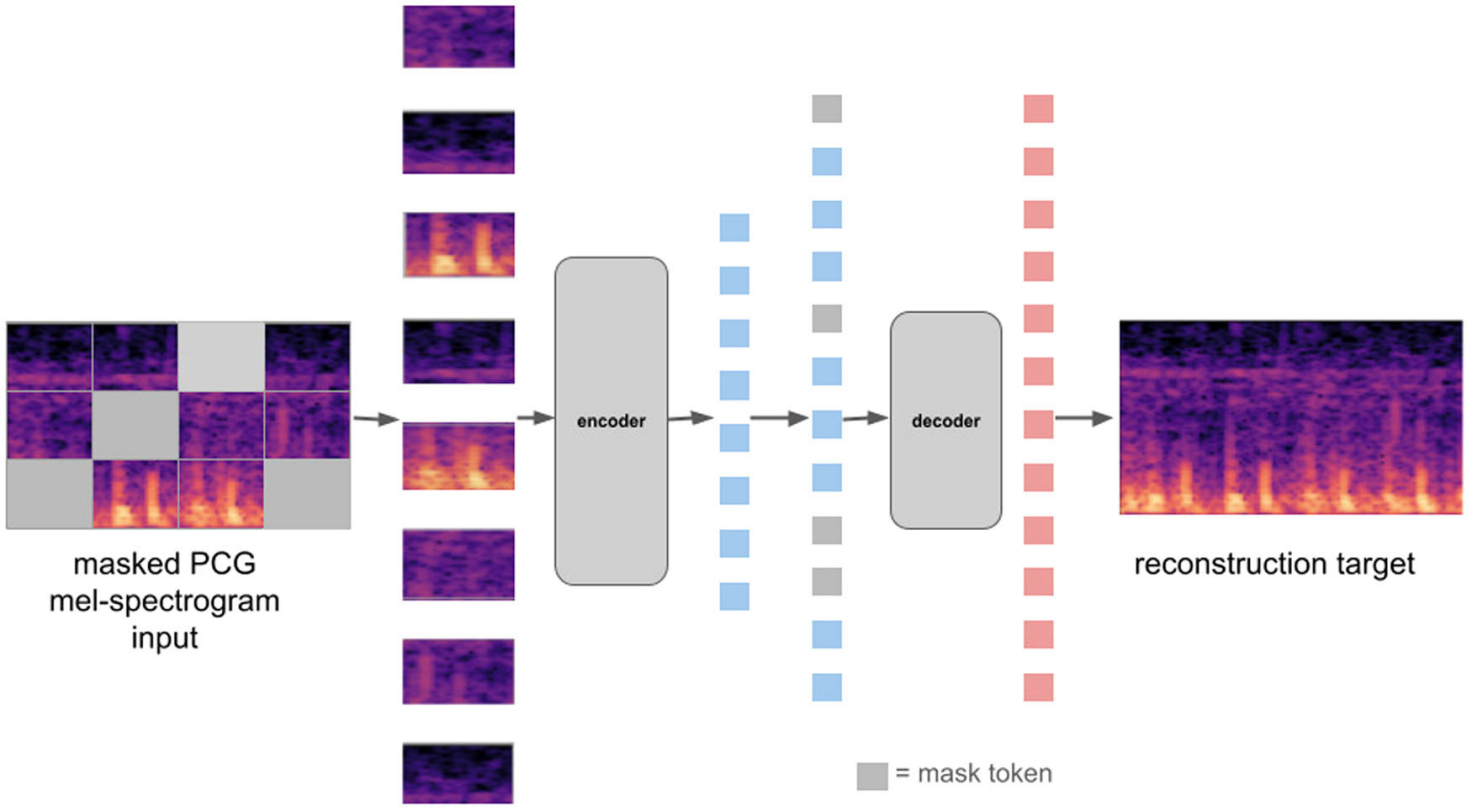
Architecture for masked autoencoder pre-training with PCG signals. The raw PCG signal is first converted to a mel-spectrogram representation (similar to what is performed for speech analysis). The mel-spectrogram is divided into regular non-overlapping patches. During pre-training, a large random subset of the mel-spectrogram patches (e.g., 70%) is masked out. The encoder component is applied on the small subset of visible patches. The latent representations of the visible patches (output of the encoder) are then unpacked and mask tokens are introduced in place of the patches that are masked out. The encoded patches and mask tokens then feed as input to the decoder component which reconstruct the original mel-spectrogram in pixels.

**Fig. 2:**
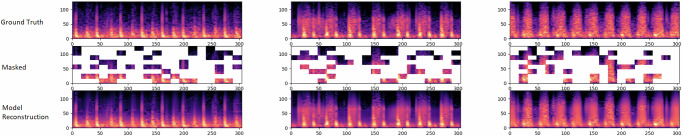
Reconstructions of masked PCG mel-spectrograms for random samples in a test set. For each triplet, we show the original mel-spectrogram (top), the masked mel-spectrogram (middle) and the reconstructed mel-spectrogram (bottom). As can be seen, the reconstructed mel-spectrograms are very close to the original ground-truth spectrograms demonstrating the model’s capability to predict the positions of the sounds in a cardiac cycle based on very limited clues from the masked mel-spectrogram. The masking ratio is 70%.

For PCG analysis, we work with its mel-spectrogram representation. Before computing the mel-spectrogram, on the raw audio, we apply a Butterworth low pass filter (with cutoff frequency 800 Hz) and a Butterflow high pass filter (with cutoff frequency 30 Hz). The mel-spectrogram is a representation of the frequency content (or spectrum) of the heart sound signal as it varies over time and which uses a mel-scale for the frequency axis to map the frequencies to perceptually meaningful bins. The usage of mel-spectrograms in PCG classification tasks has been shown to outperform the usage of the raw PCG signal^9^. The default choice of mel-spectrogram parameters we have used for our model are listed in Table 3. Figure 1 shows a schematic of the masked autoencoder pre-training approach for mel-spectrogram representations of PCG signals. Figure 2 shows reconstructions of mel-spectrograms for a few random samples made by the pre-trained model just from the masked representations of the original mel-spectrogram.

**Table 3:** Parameters of PCG mel-spectrogram computation

Parameters of PCG mel-spectrogram computation	
Length of FFT window	512
Number of mel-bands	128
Hop-length (number of samples between successive windows)	64
Lowest frequency (fmin)	0 Hz
Highest frequency (fmax)	800 Hz

**Analysis of positional embeddings**: For models that process only PCG mel-spectrograms, we find that using constant sinusoidal positional embeddings are sufficient. Nonetheless, it is interesting to understand what the model learns when using trainable positional embeddings. For this, we run an experiment where we make the positional embeddings trainable and after pre-training, we look at the similarities of the positional embeddings across the different patch positions.

**Fig. 3:**
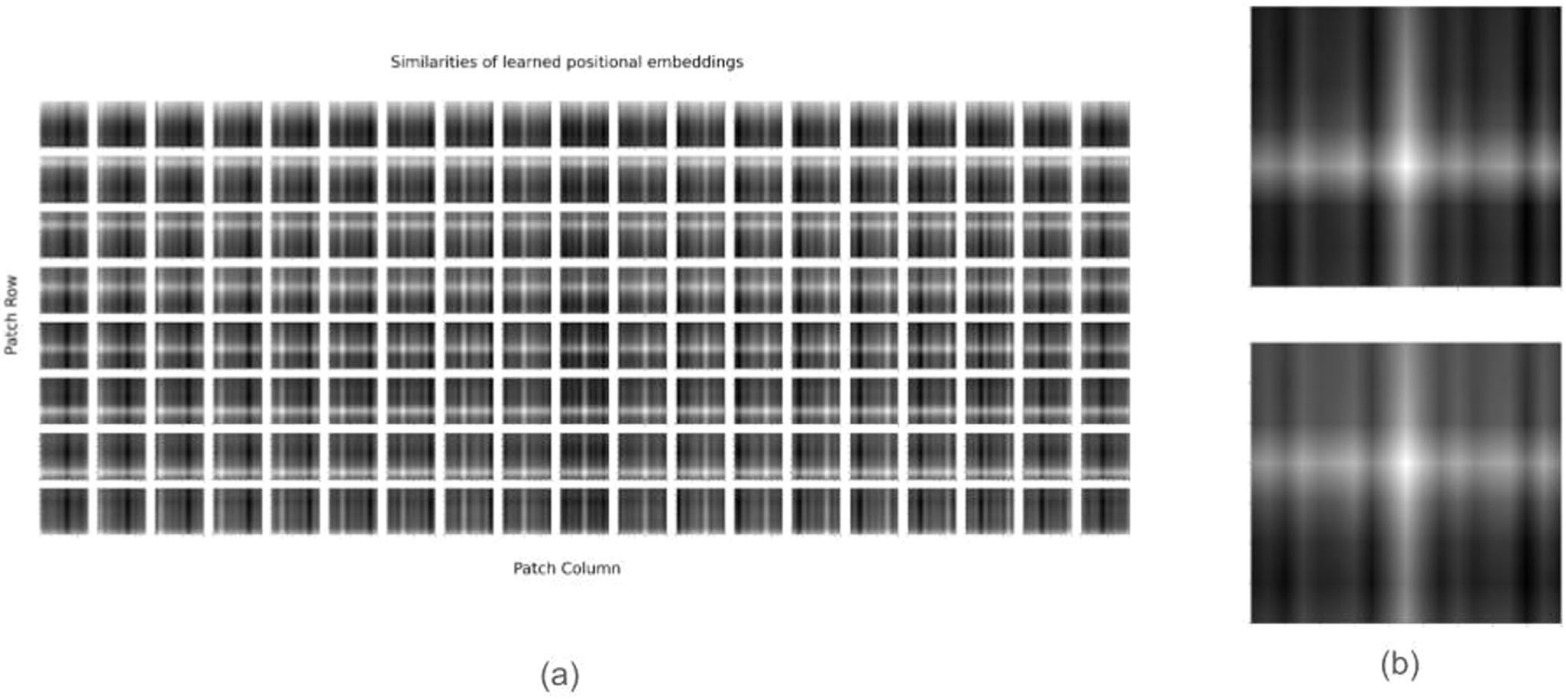
Similarities of learned position embeddings for PCG model. On the left (**a**), we show the similarities of learned position embeddings for the masked autoencoder model that analyzes PCG mel-spectrograms. Each tile shows the cosine similarities between the position embedding of the patch for the indicated row and column and the position embeddings of all other patches. On the right (**b**), we show a close-up view of the cosine similarities for two patches at the center of the PCG mel-spectrogram. The (row, column) values for the two patches are (3, 9) and (4, 9).

Figure 3 shows the similarities of the learned positional embeddings across all patches of the PCG mel-spectrogram. The results we see are similar to what was observed in the original Vision Transformer paper (See Fig. 7 of ref. ^18^), but with a couple of interesting twists. As in ref. ^18^, the model learns to encode distance between the patches of the spectrogram - closer patches tend to have similar position embeddings. Additionally, patches for a time index (a column in Fig. 3), also tend to have similar positional embeddings, meaning that the model automatically learns the time-alignment of patches originating from a spectrogram. Additionally, one can observe some periodicity along the time-axis (columns) of the cosine similarities. To emphasize this, we show a close-up view of the cosine similarities for two patches at the center of the PCG mel-spectrogram. One can see that a patch is “positionally similar” to other patches that are apart from it along the time-axis by a distance equal to multiples of a certain period. We hypothesize that this arises because the model is trained on highly periodic signals originating from the heart - thereby the model is learning that a patch in a specific phase of a cardiac cycle (or heart beat) is “positionally similar” to patches from the same phase of neighoring cardiac cycles (or heart beats). And the periodicity seen in the cosine similarities corresponds to the “average heart rate” of humans observed in the dataset. Previous use cases and demonstrations of learned positional embedding plots have not used periodic data. As such, we believe this is a novel finding that adds interpretability to what the model is learning.

#### Fine-tuning for detection of murmurs caused by SHD

Structural Heart Disease (SHD) is a leading cause of morbidity and mortality worldwide and cardiac murmurs are a common indicator of SHD. Cardiac murmurs caused by SHD are referred to as structural murmurs and those that are not related to SHD are referred to as innocent murmurs. Here we demonstrate how a PCG-based foundation model can be fine-tuned for detection of structural murmurs. Heart sound signal quality is first evaluated using a model that is trained to classify heart sound recordings as either poor signal or good signal quality (see ref. ^8^ for details). Heart sound recordings classified as good signal quality are further analyzed by the murmur classification algorithm described here. The murmur classification algorithm is trained to classify good quality heart sound recordings as having either structural murmur or no structural murmur.

**Datasets and ground truth**: The fine-tuning dataset was acquired from a combination of multisite clinical studies and from the proprietary Eko cloud database. Extensive demographic data and echocardiogram results are available for a significant subset of the recordings. This results in a fine-tuning dataset of 37,863 unique heart sound recordings (To get a sense of the scale of dataset sizes, for purely supervised training of vision-transformer models^18^, millions of labeled images are required to obtain superior performance. For our experiments, due to the initial self-supervised pre-training phase, the scale of the dataset size required for the supervised fine-tuning is relatively lower by orders of magnitude). For validation, we use a dataset of 2375 recordings from 615 unique patients. This dataset is referred to as the *SHD Murmur Validation dataset* and is described in more detail in ref. ^8^. All heart sound recordings are 5 s long and collected using the Eko CORE or Eko DUO digital stethoscopes. Patients were recruited as part of a multicenter observational study and recordings were made by research assistants trained in auscultation. All patients underwent echocardiography for gold standard evaluation and all echocardiograms were conducted with 28 days of the stethoscope recordings. A test set referred to as the *SHD Murmur Real World Evidence (RWE) dataset* was also created consisting of 2087 recordings from 368 unique patients across three different primary care clinics (See ref. ^47^ for more details). For the *SHD Murmur RWE dataset*, the patients had no prior SHD diagnosis or history of murmur. On all the datasets, a ground truth label of structural murmur is assigned to a heart sound if an audible murmur is indicated by a panel of expert annotators and the corresponding echocardiogram from the patient confirms the presence of SHD (mild or higher in severity). The sets of patients used to create the recordings for the fine-tuning dataset, the *SHD Murmur Validation dataset* and the *SHD Murmur RWE dataset* have no overlap.

**Fig. 4:**
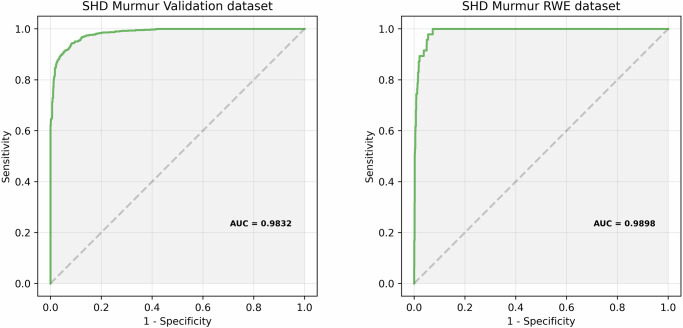
ROC curves for SHD Murmur Detection. ROC curves of the fine-tuned model for the detection of structural murmurs on the *SHD Murmur Validation dataset* and the *SHD Murmur RWE dataset*.

**Structural murmur classification results**: A model pre-trained on PCG mel-spectrograms (see Methods section) is fine-tuned on the fine-tuning dataset. Figure 4 shows the ROC curves of the fine-tuned model for the structural murmur classification task on the *SHD Murmur Validation dataset* and the *SHD Murmur RWE dataset*. Table 4 lists the Sensitivity, Specificity and AUC of the fine-tuned model on both datasets.

**Table 4:** Performance numbers of the fine-tuned model for Structural Murmur classification.

	Sensitivity (%)	Specificity (%)	AUC (%)
SHD Murmur Validation set	90.2	95.4	98.3
SHD Murmur RWE set	91.5	96.2	99.0

The decision threshold for the classification task is set for each dataset so that it maximizes Specificity subject to the constraint that Sensitivity ≥90%.

### ECG model

**Fig. 5:**
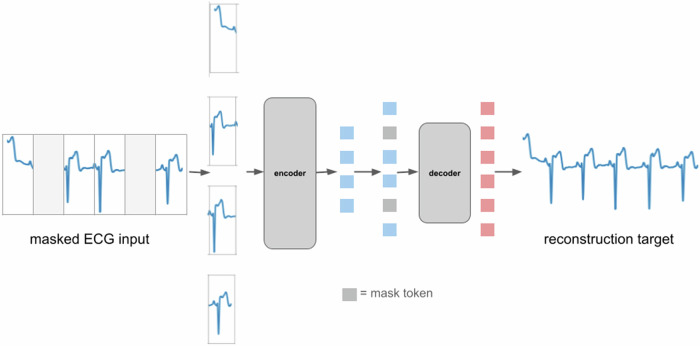
Architecture for masked autoencoder pre-training with ECG signals. The ECG signal is divided into regular non-overlapping segments. During pre-training a random subset of the ECG segments (e.g. 30%) is masked out. The encoder components acts upon the subset of visible segments. The latent presentations of the visible segments are then unpacked and mask tokens are introduced in place of the segments that are masked out. The encoded patches and mask tokens then feed as input to the decoder which reconstruct the original ECG signal sample by sample.

**Fig. 6:**
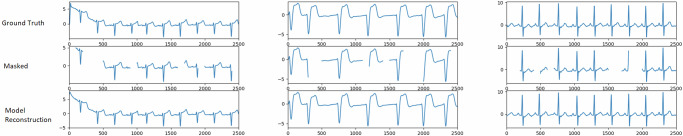
Reconstructions from masked ECG signals for random examples in a test set. For each triplet, we show the original ECG signal (top), the masked ECG signal (middle) and the reconstructed ECG signal (bottom). As can be seen, the reconstructed ECG signals are very close to the original ground-truth ECGs demonstrating the model’s capability to predict the positions of the waves in an ECG cycle based on the visible ECG segments. The length of the full ECG signal is 5 s, the segments have duration 0.2 s and the masking-ratio is 30%.

Figure 5 shows a schematic of the pre-training approach for the masked autoencoder when applied to ECG signals. Here, we directly work with the one-dimensional time-series representing the ECG trace. Before analysis, on the raw ECG signal, we apply a notch filter to remove power line noise interference at 50/60 Hz from a mains supply, a high-pass filter (with cutoff frequency 0.5 Hz) for baseline wander removal and a Savitzky–Golay filter for smoothing. We divide the ECG signal into non-overlapping segments (analogous to how PCG mel-spectrograms are divided into non-overlapping patches). For illustrative purposes, in Fig. 6, we show reconstructions of ECG signals for three random test examples made by a pre-trained model from the masked representations of the original ECG signals. For this illustration, the ECG signals have duration 5 s, the non-overlapping segments have duration 0.2 s and the masking-ratio is 30%. It is notable that the pre-trained model is capable of predicting both the periodic, stereotypical shape of the ECG waveform as well as the variation caused by baseline wander induced by the dry electrodes for ECG capture. Additionally, for the test examples shown, the ECG morphologies are quite different and yet the pre-trained model is capable of recreating the masked ECG segments based on the neighboring visible ECG segments.

**Table 5:** Performance numbers of the fine-tuned model for detection of Atrial Fibrillation.

	Sensitivity (%)	Specificity (%)	AUC (%)
AFib Validation dataset	90.2	94.9	98.0
AFib Test dataset	90.2	96.5	97.9

The decision threshold for the classification task is set for each dataset so that it maximizes Specificity subject to the constraint that Sensitivity ≥90%.

**Fig. 7:**
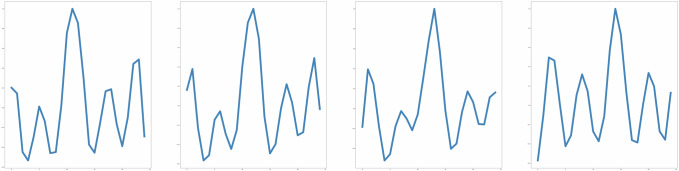
Similarities of learned position embeddings for the masked autoencoder model that analyzes ECG signals. Each trace shows the cosine similarities between the position embedding of a segment and the position embeddings of all other segments. For convenience, we show this only for four of the central segments of the ECG. The secondary peaks in each trace corresponds to the periodicity observed in the training data and indicates that a segment is “positionally similar” to other segments that are apart from it by a duration equivalent to an average beat duration.

As with PCG data, we look at trainable positional embeddings for ECG data. Figure 7 shows the similarities of the learned positional embeddings for a few of the central segments of the ECG with that of all segments of the ECG signal. Similar to what we saw for PCG data, we observe periodicity along the time-axis in the similarities which we believe arises from the fact that the model is trained on highly periodic data originating from the heart.

#### Fine-tuning for detection of Atrial Fibrillation

Atrial Fibrillation (AFib) is the most common arrhythmia and is associated with an increased risk of stroke, heart failure and other cardiovascular outcomes. Early detection of atrial fibrillation using a digital stethoscope during routine clinical visits can lead to better clinical outcomes^48,49^. We demonstrate how an ECG-based foundation model can be fine-tuned for detecting atrial fibrillation. For this task, we use a model that is pre-trained on ECG recordings of duration 15 s and where the non-overlapping segments have duration 1.25 s and the masking-ratio used for pre-training is 30%. The fine-tuning task is a rhythm classification task with four rhythm classes: Normal Sinus Rhythm, Atrial Fibrillation, Noise and Other. The Other rhythm class can be thought of as consisting cardiac arrhythmias like supraventricular tachycardia or frequent ectopy which can be challenging to distinguish from Atrial Fibrillation. This labeling scheme is consistent with previous literature and was specified in the 2017 Physionet Challenge data (https://physionet.org/content/challenge-2017/1.0.0/).

**Datasets and ground truth**: The fine-tuning dataset was created using recordings in the proprietary Eko cloud database collected using the Eko DUO device. The fine-tuning dataset consists of 7997 unique single-lead ECG recordings. The validation and test sets were also created using recordings in the Eko cloud database collected using the Eko DUO device. The *AFib Validation dataset* consists of 2002 unique single-lead ECG recordings and the *AFib test dataset* consists of single-lead ECG recordings from 732 unique patients. For the fine-tuning dataset and the *AFib Validation dataset*, the ground truth was established by a panel of trained annotators using the CentaurLabs annotation platform (https://www.centaurlabs.com/). For the *AFib Test dataset*, the ground truth was established by a panel of of three cardiologists who analyzed the rhythm of each recording independently. In any case where there was a disagreement between the three cardiologists, the majority opinion was taken as the ground truth. The sets of patients used to create the recordings for the fine-tuning dataset, the *AFib Validation dataset* and the *AFib test dataset* have no overlap.

**Fig. 8:**
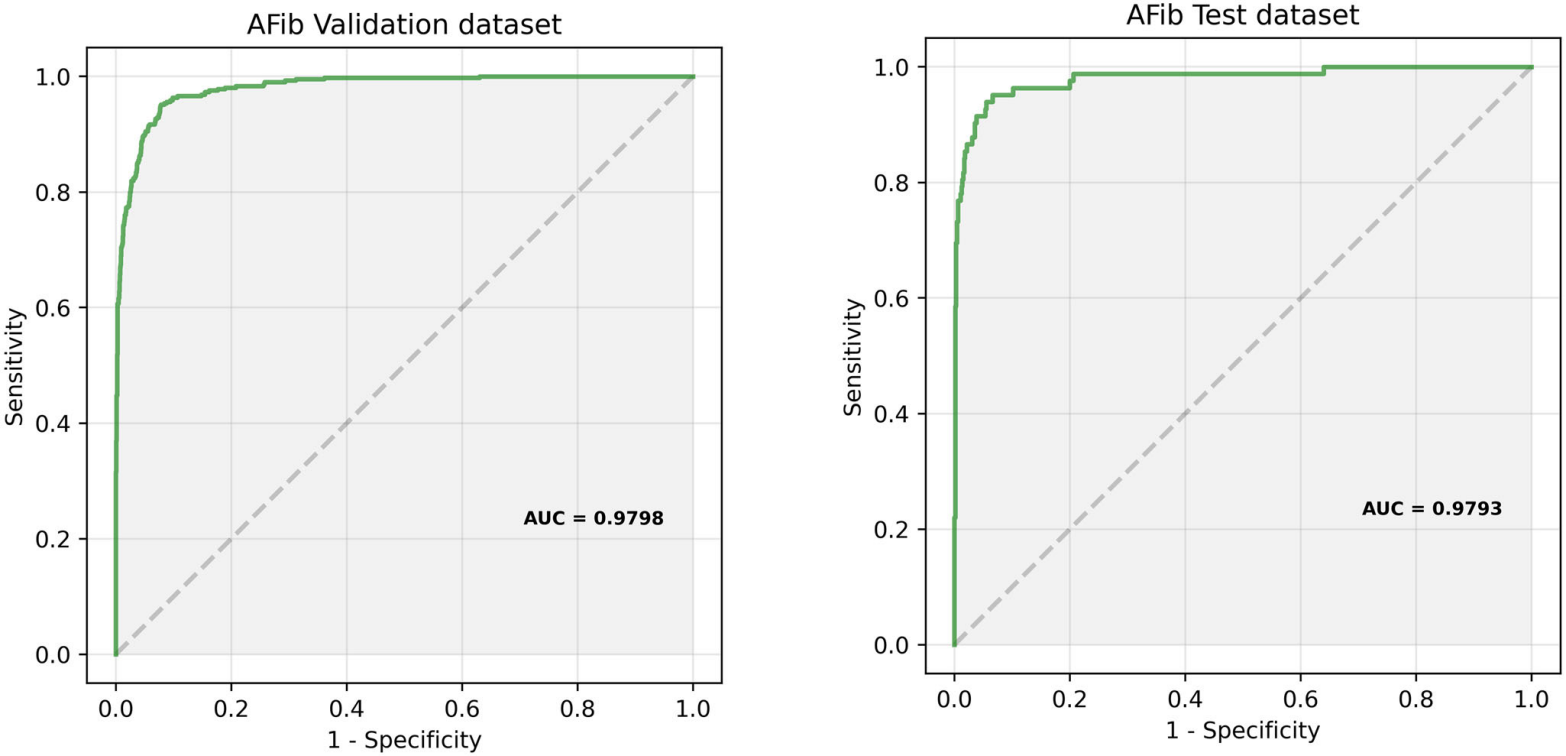
ROC curves of the fine-tuned model for the detection of Atrial Fibrillation on the AFib Validation dataset and the AFib Test dataset. The optimal fine-tuned model parameters are specified in Table 8.

**Atrial fibrillation detection results**: A model pre-trained on ECG signals (see Methods section) is fine-tuned on the fine-tuning dataset. Figure 8 shows the ROC curves of the fine-tuned model for the detection of Atrial Fibrillation on the *AFib Validation dataset* and the *AFib Test dataset*. Table 5 lists the Sensitivity, Specificity and AUC of the fine-tuned model for the detection of Atrial Fibrillation on both datasets.

### Model for synchronous PCG and ECG

**Fig. 9:**
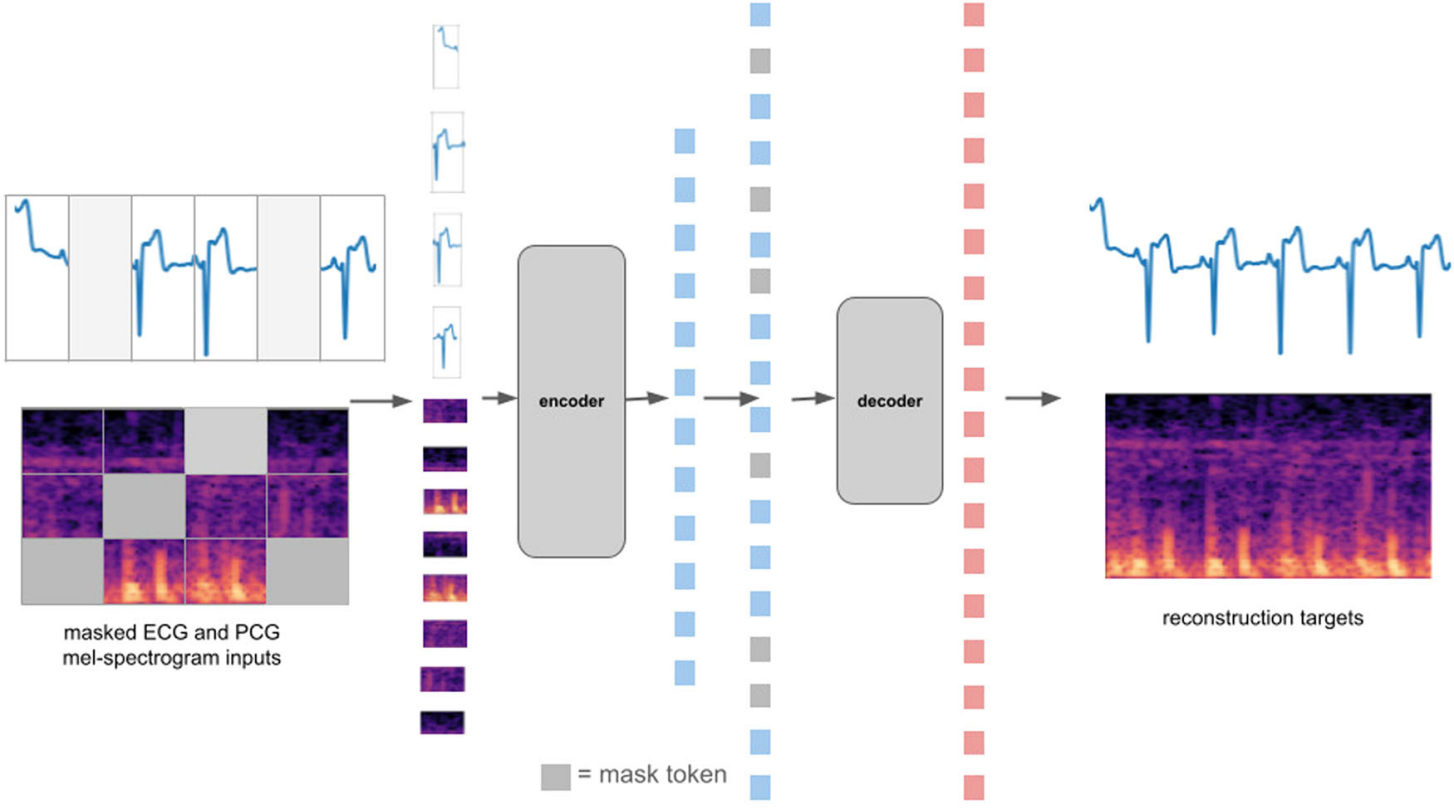
High-level architecture for masked autoencoder pre-training with multiple signals (ECG and PCG). The Masked AutoEncoder model is adapted to process synchronously captured ECG and PCG signals. Pre-training models in this fashion allows the model to learn patterns in the timing of electrical activites of the heart relative to the timing of mechanical activities of the heart (as manifested in the heart sounds via PCG). Thereby, in addition to features from the individual ECG and PCG signals, features related to relative timings of electrical and mechanical activity are also learned by the model which can be useful to detect people in disease states.

A powerful capability that is made possible with digital stethoscopes that capture both PCG and ECG (https://www.ekohealth.com/products/core-500-digital-stethoscope) is the creation of models that can analyze synchronous PCG and ECG jointly. Training on synchronous PCG and ECG signals allows the model to learn relationships across both signals. To enable this, we have extended the approach described in the previous subsections to incorporate multiple signals that are synchronously captured. These models learn subtle connections across the different physiological signals (e.g. PCG and ECG) and thus be able to make more refined predictions for downstream classification tasks. Figure 9 shows a schematic of the pre-training of the masked autoencoder when working with multiple synchronous signals (PCG and ECG).

**Fig. 10:**
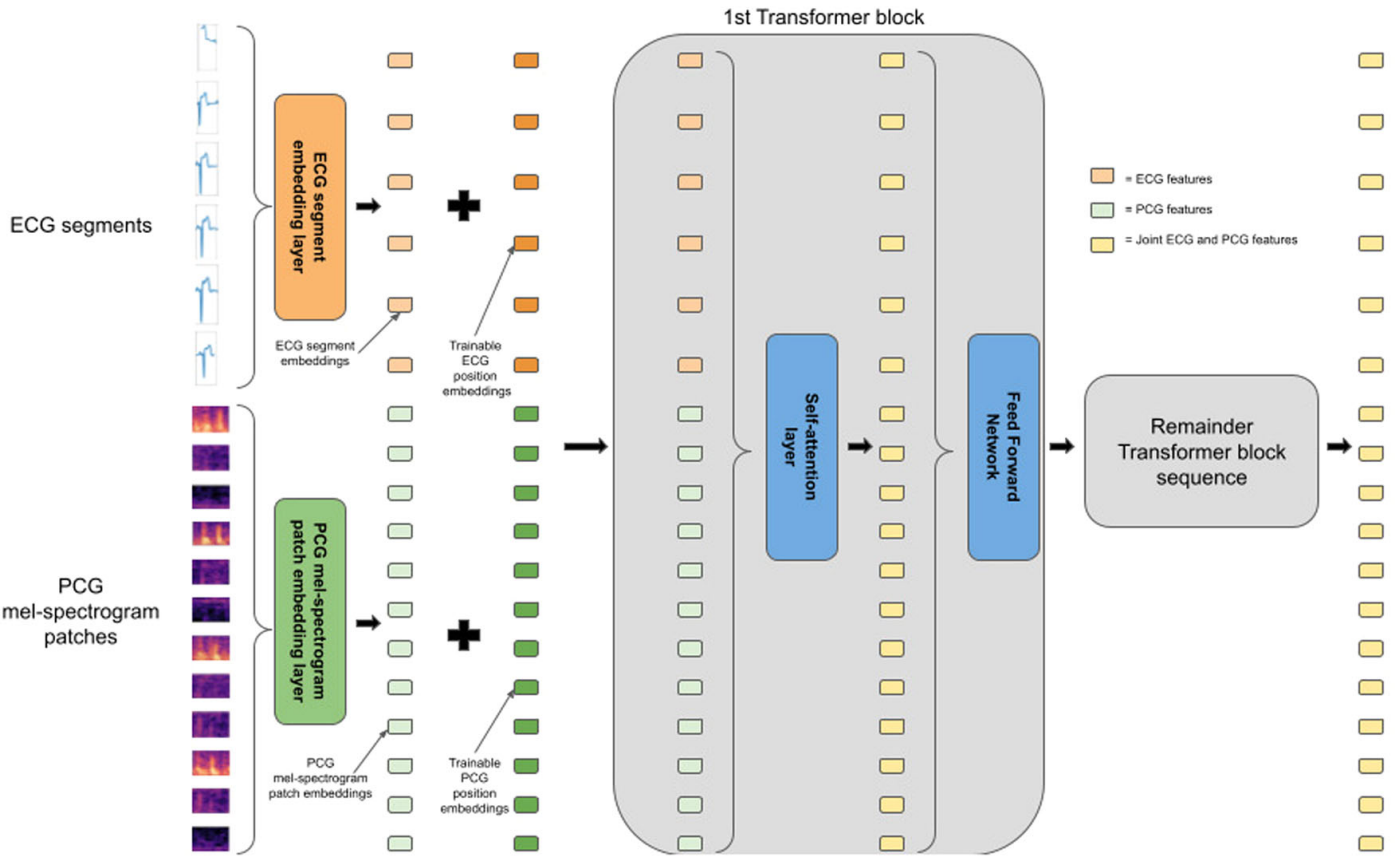
Closer look at the encoder component of the model for synchronous PCG and ECG. This illustration is shown for the case where there is no masking. First, the ECG segments and PCG mel-spectrogram patches are processed by their respective embedding layers. Both embedding layers produce embeddings of the same dimension. The ECG segments and PCG mel-spectrogram patches have trainable position embeddings (also of the same dimension) for each position which are added on to the respective segment/patch embeddings. After the addition of the trainable position embeddings, the resulting features feed into the first transformer block. Within the self-attention layer of the first transformer block, ECG features and PCG features at all positions interact with each other to produce joint ECG and PCG features at each position which then feed into the feed-forward network and then the remaining sequence of transformer blocks (for brevity, residual connections and normalization steps are not displayed here).

Similar to the models that analyze individual signals, the multi-signal variant of the model has a single encoder. The main difference is that the encoder has separate embedding layers for the ECG segments and patches of the PCG mel-spectrogram. Also, both the ECG and PCG mel-spectrogram have their own trainable positional embeddings which are added onto the segment/patch embeddings. For a closer look at the implementation of the encoder for the multi-signal variant of the model, see Fig. 10. As can be see in Fig. 10, by design, within the transformer layers of the encoder, the tokens corresponding to the ECG segments and the PCG mel-spectrogram patches interact with each other via the attention mechanism. Hence, the latent representations generated by the encoder capture features not just from the individual ECG and PCG signals alone, but also features related to the relative timing of electrical activities and mechanical activities of the heart.

**Fig. 11:**
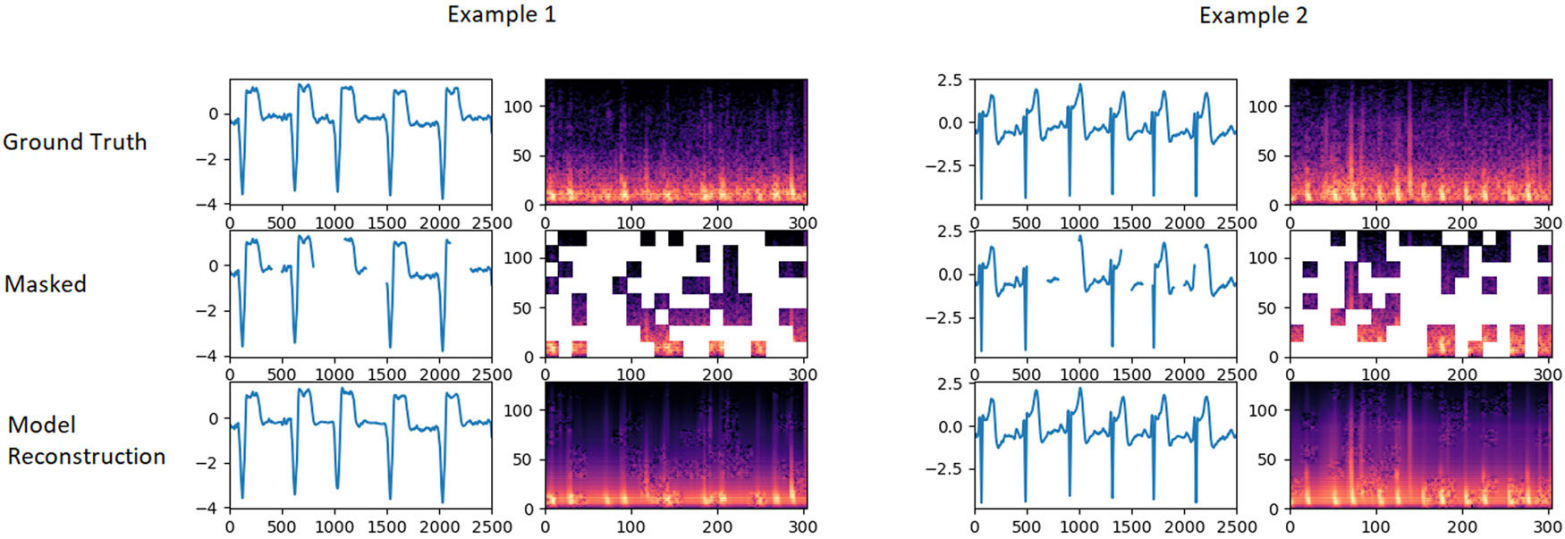
Example reconstructions for synchronous PCG and ECG data. For each example, we show the original ECG signal and PCG mel-spectrogram (top), the masked ECG signal and PCG-mel-spectrogram(middle) and the reconstructed ECG signal and PCG mel-spectrogram(bottom). The length of the signals are 5 seconds, the masking-ratios for ECG and PCG are 30% and 70% respectively.

The multi-signal variant of the model also has a single decoder. The latent representatons of the visible (non-masked) ECG segments and patches (of the PCG mel-spectrogram) generated by the encoder are unpacked and mask tokens are inserted in place of the segments/patches that are masked out. Note that the same, shared, learned vector is used as a mask token in place of both ECG segment and patches (of the PCG mel-spectrogram) that are masked. Since the encoder outputs jointly represent the ECG and PCG signals, for reconstruction of the original ECG signal, the decoder indirectly uses features of the PCG signal and vice-versa. Separate linear prediction layers are used to reconstruct the original ECG signal and PCG signals. Figure 11 shows sample reconstructions for synchronous PCG and ECG data.

#### Fine-tuning for detection of Low Ejection Fraction

Low Ejection Fraction (EF) is evidence of heart failure and is associated with high mortality rates. Echocardiography is the gold standard for diagnosing low EF, but its effectiveness is limited by resource constraints, the need for trained personnel and cost. A noninvasive and low-cost screening tool for low ejection fraction can lead to more early interventions and thus improve health outcomes. This has led to attempts by groups to develop tools that use ECG for early detection of low ejection fraction^14,50^. Here, we describe a model that uses PCG and ECG signals from a digital stethoscope for the detection of low EF.

**Datasets and ground truth**: The Low EF fine-tuning dataset consists of 11,067 synchronous PCG and ECG signals captured using the Eko DUO device. The *Low EF Validation dataset* consists of synchronous PCG and ECG signals captured from 2960 unique patients (this dataset is the same as described in detail in ref. ^50^). For both the fine-tuning and validation datasets, all patients underwent echocardiography within a maximum of seven of days of the corresponding recordings with the digital stethoscope. The true EF of each patient was measured by the echocardiogram machine’s integrated cardiac quantification software at the time of the echocardiogram. The EF measurements were then over-read by board-certified cardiologists at each site using Simpson’s biplane method. The EF status of each patient is categorized as Low EF (≤40%) or Normal EF (>40%). There is no overlap of patients across the Low EF fine-tuning dataset and *Low EF Validation dataset*.

**Fig. 12:**
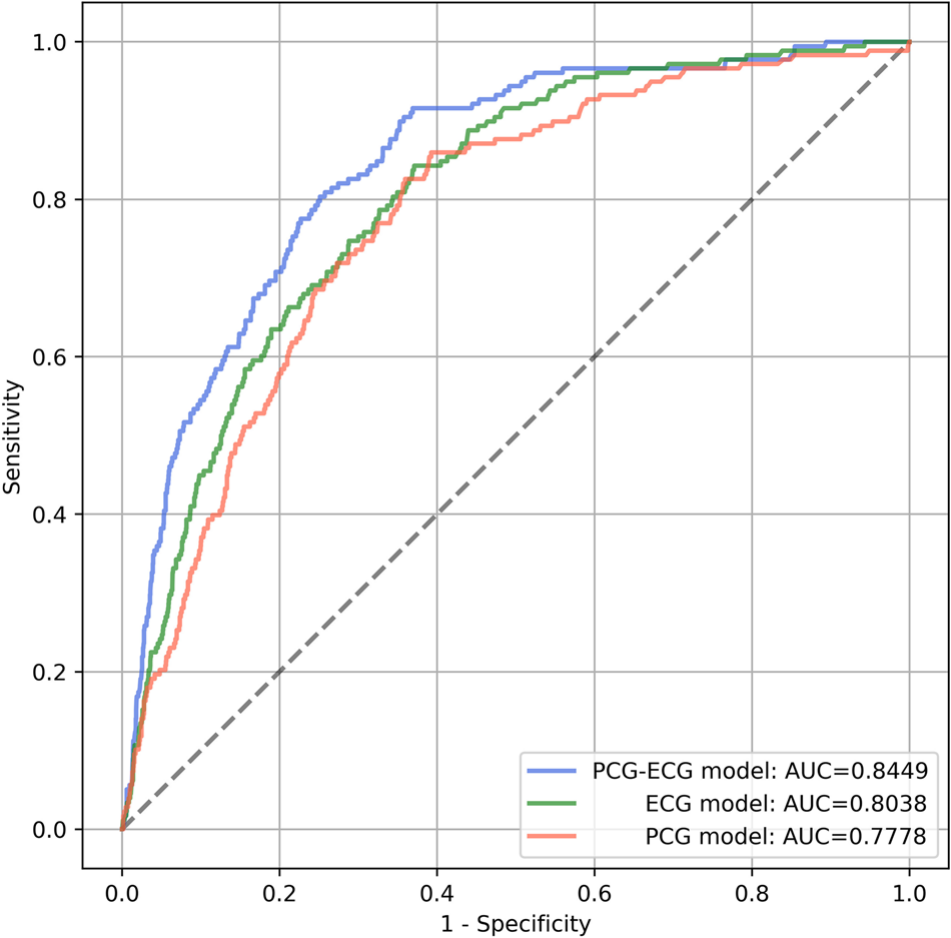
ROC curves of fine-tuned models for the detection of Low Ejection Fraction on the *Low EF Validation dataset.* Results are shown for the model that analyzes synchronous PCG and ECG simultaneously (PCG-ECG model), as well as for the models that analyze ECG and PCG individually (ECG-model and PCG-model).The optimal fine-tuned model parameters are specified in Table 8.

**Low Ejection Fraction detection results**: A model pre-trained on synchronous PCG and ECG signals (see Methods section) is fine-tuned on the Low EF fine-tuning dataset. The fine-tuning task is a binary classification task that takes in a synchronous pair of PCG and ECG signals and returns a binary value indicating whether the patient has low EF (whose ground-truth is determined using echocardiography). The ROC curves for the detection of Low EF on the *Low EF Validation dataset* are shown in Fig. 12. In addition to the ROC curve for the PCG-ECG model that analyzes synchronous PCG and ECG signals, we also show the ROC curves for separate models that analyze the PCG and ECG signals individually. These individual signal models are pre-trained and fine-tuned using the exact same masked autoencoder framework and hyperparameters as for the PCG-ECG model, but took only one of the signals as input. Table 6 lists the Sensitivity, Specificity and AUC on the *Low EF Validation dataset* for the fine-tuned PCG-ECG model as well as the individual signal models. The decision threshold for the classification task is set for each model so that it maximizes Specificity subject to the constraint that Sensitivity ≥75%.

**Table 6:** Performance numbers of the fine-tuned models on the *Low EF Validation dataset* for detection of Low Ejection Fraction.

	Sensitivity (%)	Specificity (%)	AUC (%)
PCG-ECG model	75.3	78.3	84.5
ECG model	75.3	70.0	80.4
PCG model	75.3	68.2	77.8

The decision threshold for the classification task is set for each model so that it maximizes Specificity subject to the constraint that Sensitivity ≥75%.

As can be seen from Fig. 12 and Table 6, the PCG model has the lowest AUC indicating that PCG is the least predictive input for Low EF. The ECG model has the next highest AUC indicating that ECG inputs have more features that are indicative of Low EF compared to PCG inputs. The PCG-ECG model has the best AUC and this result suggests that the PCG-ECG model is successfully utilizing features related to the relative timing of electrical and mechanical activities of the heart for detecting patients that have low EF. We believe that the performance of the PCG-ECG model can get better by having more ECG leads in the input data and with larger fine-tuning datasets. For models of the size that we currently use, the size of the fine-tuning dataset is a factor that limits the number of layers in the encoder component that can be unfrozen during the fine-tuning phase. With larger fine-tuning datasets, we could potentially unfreeze more transformer layers of the encoder and attain even better performance (For inference on one sample with this model, on the AMD EPYC 7J13 CPU with 64 cores, it takes approximately 195 ms, whereas on the NVIDIA A100 GPU, it takes approximately 6 ms.).

## Discussion

We have demonstrated the paradigm of foundation models being applied for bio-signals (PCG and ECG) captured using digital stethoscopes from patients during regular clinical practice. As examples, we showed these foundation models being fine-tuned for detection of murmurs caused by SHD, atrial fibrillation and low ejection fraction. Our earlier work has shown that the masked autoencoder approach outperforms convolutional neural networks (CNNs) for the detection of murmurs caused by SHD^51^. A systematic comparison of this new approach against CNN-style models for all the tasks described in this paper will be the subject of future work.

Obviously, the application of this new paradigm is not limited to the detection of cardiovascular conditions detailed in this paper. It can be readily applied for a wide variety of detection tasks - some of them being pulmonary hypertension, S3 and S4 heart sounds (which are indicative of heart failure), hypertrophic cardiomyopathy, heart block etc. The only limiting factor to developing models for detecting these abnormalities is a reasonably-sized labeled dataset to facilitate the fine-tuning of the foundation models. We additionally present and demonstrate the benefit of using a multi-signal transformer framework on synchronized bio-signals. This framework can be easily generalized for many applications including 12-lead ECG analysis and Early Warning Scores (EWS) generated by bedside monitors (e.g. ref. ^52^).

The paradigm described here also readily applies to analysis of lung sounds captured from digital stethoscopes (for e.g., detection of wheeze, crackle and rhonchi). This approach has been demonstrated for cough and breath sounds by a team at Google Research^46^ and for detection of COVID-19, tuberculosis and characteristics such as whether a person smokes.

In terms of future research directions, one interesting goal would be to make the results of the fine-tuned models more explainable to the end-user. This is a vast research domain in the field of deep learning and explainability is an especially important goal for clinical applications to be able to gain the trust of clinicians. One approach to help with explainability is the generation of saliency maps that can be visualized by the end-user. Saliency maps are auxiliary outputs of the model that highlight regions or segments of the input signal that are most relevant for its classification. Various methods to generate saliency maps for vision transformer models have been explored. There are attention-based methods^53^, gradient-based methods^54,55^ and perturbation-based methods^56–58^. Combining one of these saliency map approaches with our masked autoencoder models will be the subject of future work.

In addition to saliency map generation, in the future, one can envision models that not only provide classification results based on bio-signals, but also provide textual descriptions to the clinician for why the model made a certain prediction. This is possible with GPT-style models (https://openai.com/index/gpt-4o-mini-advancing-cost-efficient-intelligence/) that are capable of multimodal reasoning and one can fine-tune (https://cookbook.openai.com/examples/how_to_finetune_chat_models) such models by providing datasets of image representations of bio-signals paired with textual descriptions of what they indicate. There are obvious challenges with these type of approaches given the risk of the model generating “hallucinatory” outputs which are inconsistent with the input data. More research is needed to be able to use such models in a safe and reliable manner.

In summary, we believe that the paradigm of foundation models is a necessity for bio-medical applications because of the enormous challenges related to creating high-quality datasets with reliable medical label annotations. The results in this paper show that if unlabeled data sources are available, one should utilize them to build large capacity foundation models which can then be fine-tuned on a relatively smaller-sized labeled dataset for disease detection tasks.

## Methods

The masked autoencoding approach we use is inspired by the work in ref. ^5^. We pre-train models to reconstruct the original signals (ECG and/or PCG) given their partial observations (masked representations). The approach has an encoder that maps the observed signal to a latent representation, and a decoder that reconstructs the original signal from the latent representation. An asymmetric design is used that allows the encoder to operate only on partial, observed signals and a lightweight decoder that reconstructs the full signals from the latent representation and mask tokens. After pre-training, the decoder is discarded and the encoder is applied to the complete, unmasked signals to obtain features that feed into a classification head. In this work, we describe models that work individually on PCG/ECG signals as well as models that work on synchronously captured PCG and ECG signals.

Before the analysis of PCG signals and ECG signals, we apply digital filters on them as listed in Table 1. When working with PCG signals, we convert the filtered audio signal to a mel-scaled, decibel (dB)-scaled spectrogram (mel-spectrogram) representation for analysis by the model. The mel-spectrogram representation is the dominant type of representation used in deep learning for audio signal processing. For a review, see ref. ^59^. The usage of mel-spectrogram representations is motivated by the fact that the mel-scale is designed to approximate how humans perceive sound. Humans perceive differences in low frequency ranges more easily compared to differences in high frequency ranges. This makes mel-spectrograms more aligned with how humans perceive audio and thus making them a natural choice for audio analysis tasks. The mel-spectrogram also reduces the number of frequency bins compared to a linear scale, leading to a lower-dimensional representation and thereby making processing by deep-learning models more computationally efficient and reducing the risk of overfitting. The efficacy of mel-spectrogram representations derived from PCG signals for heart sound classification has already been demonstrated in refs. ^7,10^ and ref. ^8^ and motivated by this, for this study, we also work with mel-spectrogram representations of PCG signals. Models that work directly with raw audio waveforms and where the initial layers of the deep learning system are designed and trained to extract customized features similar to those for mel-spectrogram representations will be the subject of future work. For prior work in this direction, see refs. ^60–63^ and ref. ^64^.

### Masking of ECG and PCG representations

The PCG signals are converted to mel-spectrograms and treated as single-channel images. We work directly with ECG signal as a one-dimensional time-series. The mel-spectrogram representation of the PCG is divided into regular non-overlapping patches. Similarly, the ECG signal is divided into non-overlapping segments. Random patches of the PCG mel-spectrogram and random segments of the ECG are sampled without replacement following a uniform distribution. The masking ratios (i.e., the ratio of removed patches/segments) used for the PCG and ECG representations are different and are optimized.

### The encoder component

The encoder component is a Vision Transformer model but is applied only on the visible, unmasked patches (of the PCG mel-spectrogram) and/or segments of the ECG. For the individual signal models, similar to the standard ViT, the encoder embeds patches (or segments) with a linear projection and then adds positional embeddings to them. The resulting embedded patches (or segments) are then processed by a series of Transformer blocks. For the individual signal models, we use the standard sinusoidal positional embeddings as in ref. ^15^ and observe that similar to ref. ^15^ and ref. ^18^, we obtain nearly identical results when using trainable positional embeddings.

For the multi-signal variant of the model that analyzes synchronously captured PCG and ECG signals, the encoder has separate linear projection layers for the patches of the PCG mel-spectrogram and segments of the ECG. Additionally, the PCG patches and ECG segments have their own trainable positional embeddings. After the positional embeddings are added to the embedded patches and segments, they are processed simultaneously by one series of Transformer blocks. This design allows the PCG mel-spectrogram patches and ECG segments to interact with each other via the attention mechanism and thus the model learns features coming from the individual signals as well as relationships between PCG and ECG features.

### The decoder component

The encoder components processes the visible, unmasked patches/segments to provide their latent representations. These latent representations or encoded patches/segments and mask tokens then feed as inputs to the decoder component (See Figs. 1 and 9). As in ref. ^2^ and ref. ^5^, each mask token is a shared, learned vector that indicates the presence of a missing patch/segment to be predicted. Just as for the encoder component, for the multi-signal variant of the model, the tokens originating from PCG and ECG signals have their own trainable positional embeddings. For models working with PCG/ECG signals individually, by default, we use the standard sinusoidal positional embeddings. After adding the positional embeddings to the tokens, they are processed by a series of Transformer blocks.

The decoder component has final linear prediction layers which take the output of the sequence of Transformer blocks and make predictions for the masked patches and/or segments. For the individual signal models, there is a single linear prediction layer for reconstructing the masked patches (or segments). For the multi-signal variant of the model, separate linear prediction layers are used for the tokens corresponding to the PCG and ECG signals to reconstruct the PCG mel-spectrogram patches and ECG segments respectively. The decoder component is used only during the pre-training phase to perform the reconstruction task for the PCG mel-spectrograms and/or the ECG signal. As per ref. ^5^, the decoder component is typically lightweight compared to the encoder component and is therefore narrower and shallower than the encoder.

### Reconstruction task

The Masked AutoEncoder model reconstructs the input signals by predicting the pixel values for each masked patch of the PCG mel-spectrogram and/or the sample values for each masked segment of the ECG signal. Each element in the decoder’s output is a vector of pixel (or sample) values representing a patch (or segment). As discussed earlier, the final linear prediction layers of the decoder component produces an output whose number of channels equals the number of pixel (or sample) values in a patch (or segment). The decoder’s outputs are reshaped to form the reconstructed PCG mel-spectrograms and/or ECG signal. The loss function computes the mean squared error (MSE) between the reconstructed signals and original signals in the pixel (or sample) space and this MSE is summed over the PCG and ECG signals for the multi-signal variant of the model. The loss is computed only on the masked patches (or segments) as for the BERT model training^2^.

### Fine-tuning task

All the fine-tuning tasks described in this paper are classification tasks. For the fine-tuning phase, the decoder component is discarded. An average pooling layer is applied on the features coming out from the encoder component and the pooled output feeds into a linear classification head whose number of outputs is equal to the number of classes for the classification task. As in ref. ^19^, for the individual PCG model, we use structured masking of the PCG mel-spectrogram during fine-tuning. Structured masking during fine-tuning amounts to masking random bands along the frequency axis or time-axis of the mel-spectrogram. For the individual ECG model and the multi-signal variant of the model, we use random masking during fine-tuning, but the masking-ratios used are lower compared to that used during pre-training. During the fine-tuning phase, only the last few layers of the encoder and the classification head are fine-tuned while freezing the initial layers of the encoder. The number of final layers of the encoder that are unfrozen is a hyper-parameter that is adjusted for each classification task.

The pre-training settings and dataset sizes used for each of the models are listed in Table 7. The range of values swept over for each hyper-parameter and the optimal values for each hyper-parameter are listed for each classification task in Table 8.

**Table 7:** Pre-training settings for all three models

	PCG model	ECG model	PCG-ECG model
Optimizer	AdamW	AdamW	AdamW
Base learning rate	1.5e−4	8e−5	1e−5
Weight decay	0.05	0.05	0.05
Optimizer momentum	*β*_1_, *β*_2_ = 0.9, 0.95	*β*_1_, *β*_2_ = 0.9, 0.95	*β*_1_, *β*_2_ = 0.9, 0.95
Batch size	2048	2048	2048
Learning rate schedule	Cosine decay	Cosine decay	Cosine decay
Warmup epochs	40	40	40
Training epochs	700	2000	5000
Pre-train dataset size	1,890,304	241,664	221,184
Signal duration	5 s	15 s	PCG: 5 secs
			ECG: 5 secs
Patch (or segment size)	16 × 16	1 × 625	PCG: 16 × 16
			ECG: 1 × 100
Masking ratio	70%	30%	PCG: 70%, ECG: 30%
Learnable positional embeddings	False	False	True

**Table 8:** Fine-tuning settings for SHD murmur detection, AFib detection and Low Ejection Fraction detection.

	SHD murmur	AFib	Low ejection fraction
Optimizer	AdamW	AdamW	AdamW
Base learning rate	1e−3	1e−3	1e−3
Weight decay	0.05	0.05	0.05
Optimizer momentum	*β*_1_, *β*_2_ = 0.9, 0.999	*β*_1_, *β*_2_ = 0.9, 0.999	*β*_1_, *β*_2_ = 0.9, 0.999
Batch size	512	512	512
Learning rate schedule	Cosine decay	Cosine decay	Cosine decay
Warmup epochs	5	5	5
Training epochs	50	50	50
Type of masking	Structured	Random	Random
Label smoothing	0.1, 0.2, 0.3^*^	0.1, 0.2, 0.3^*^	0.1^*^, 0.2, 0.3
Masking ratio	20%, 30%^*^, 40%	0%, 10%^*^, 20%, 30%	PCG: 10%^*^, 20%, 30%
			ECG: 10%, 20%, 30%^*^
Number of encoder half-blocks unfrozen	1, 2, 3^*^, 4	1, 2, 3^*^, 4	1, 2^*^, 3, 4
Best AUC on validation set	98.3%	98.0%	84.5%

For the hyper-parameters (label-smoothing, masking-ratio, number of encoder half-blocks unfrozen) we show the list of values over which we perform a sweep for each fine-tuning task and the asterisk sign highlights the values which had the best performance (AUC) on the corresponding validation set. When the number of encoder half-blocks unfrozen is equal to one, this means that only the feed-forward part of the last transformer block is unfrozen. When the number of encoder half-blocks unfrozen is equal to two, the entirety of the last transformer block is unfrozen and so forth.

## Supplementary information

CV - MI-CLAIM checklist

## Data Availability

Eko Health will consider requests to access the training datasets and test datasets on an individual basis. Any data use will be restricted to noncommercial research purposes, and the data will be made available only on execution of appropriate data use agreements.

## Code availability

The code used to implement the models described in this paper will be made available upon request.
