# Supplementary material for: Foundation models for cardiovascular disease detection via biosignals from digital stethoscopes
Source: NPJ Cardiovasc Health. 2024 Oct 11;1:25. doi: 10.1038/s44325-024-00027-5 (PMC12912441; doi:10.1038/s44325-024-00027-5)
Supplement: Supplementary file 1 — CV - MI-CLAIM checklist [file 44325_2024_27_MOESM1_ESM.pdf]

Table 1 | (From: https://www.ncbi.nlm.nih.gov/pmc/articles/PMC7538196/)

The MI-CLAIM checklist

Before paper submission

**Study design (Part 1)**	**Completed: page number**	**Notes if not completed**
The clinical problem in which the model will be employed is clearly detailed in the paper.	Yes: Page 1 (Introduction)	
The research question is clearly stated.	Yes: Page 1 (Introduction)	
The characteristics of the cohorts (training and test sets) are detailed in the text.	Yes: Page 5 and 7 (References 8, 47 and 50)	
The cohorts (training and test sets) are shown to be representative of real-world clinical settings.	Yes: Page 5 and 7 (References 8, 47 and 50)	
The state-of-the-art solution used as a baseline for comparison has been identified and detailed.	N/A	Discussed in Reference 51
**Data and optimization (Parts 2, 3)**	**Completed: page number**	**Notes if not completed**
The origin of the data is described and the original format is detailed in the paper.	Yes: Page 2 (Introduction)	
Transformations of the data before it is applied to the proposed model are described.	Yes: Pages 3 and 5.	
The independence between training and test sets has been proven in the paper.	Yes: Pages 5, 6 and 7	
Details on the models that were evaluated and the code developed to select the best model are provided.	Yes: Page 9 (Methods)	

Is the input data type structured or unstructured?	X Structured ☐ Unstructured	
**Model performance (Part 4)**	**Completed: page number**	**Notes if not completed**
The primary metric selected to evaluate algorithm performance (e.g., AUC, F-score, etc.), including the justification for selection, has been clearly stated.	Yes: Page 3.	
The primary metric selected to evaluate the clinical utility of the model (e.g., PPV, NNT, etc.), including the justification for selection, has been clearly stated.	N/A	
The performance comparison between baseline and proposed model is presented with the appropriate statistical significance.	N/A	
**Model examination (Part 5)**	**Completed: page number**	**Notes if not completed**
Examination technique 1[a]	Yes: Page 3	
Examination technique 2[a]	N/A	
A discussion of the relevance of the examination results with respect to model/algorithm performance is presented.	Yes: Page 8 (Discussion)	
A discussion of the feasibility and significance of model interpretability at the case level if examination methods are uninterpretable is presented.	Yes: Page 8 (Discussion)	
A discussion of the reliability and robustness of the model as the underlying data distribution shifts is included.	No.	To be included in a follow-up manuscript

**Reproducibility (Part 6): choose appropriate tier of transparency**		**Notes**
Tier 1: complete sharing of the code	☐	
Tier 2: allow a third party to evaluate the code for accuracy/fairness; share the results of this evaluation	☐	
Tier 3: release of a virtual machine (binary) for running the code on new data without sharing its details	☐	
Tier 4: no sharing	X	Code availability statement included in manuscript - code will be made available upon request

PPV, positive predictive value; NNT, numbers needed to treat.

[a]Common examination approaches based on study type: for studies involving exclusively structured data, coefficients and sensitivity analysis are often appropriate; for studies involving unstructured data in the domains of image analysis or natural language processing, saliency maps (or equivalents) and sensitivity analyses are often appropriate.
